# Management of drooling for patients in the north of Iran: Analysis of the surgical management

**Published:** 2010

**Authors:** Seyed Ebrahim Naghavi, Mir Mohammad Jalali

**Affiliations:** a The Drooling Clinic of Amiralmomenin Hospital, Guilan University of Medical Sciences, Rasht, Iran

**Keywords:** Drooling, Sialorrhea, Submandibular Relocation, Salivary Glands

## Abstract

**BACKGROUND:**

Drooling is a common problem in children and adults with neuromuscular disorders. This problem is best dealt with using a multidisciplinary team approach. The objective of this paper is to assess the results following surgery at the Drooling Clinic of Amiralmomenin Hospital.

**METHODS:**

The results of the surgical protocol used between 1994 and 2007 at the Drooling Clinic of Amiralmomenin Hospital in Rasht, Iran, reported thirty-two patients underwent submandibular duct relocation and sublingual resection. The preoperative and postoperative levels of drooling were measured. The parents of the patients were contacted by telephone at least one year after operation.

**RESULTS:**

Of all the patients, eighteen were male and fourteen were female and were aged 6 years to 26 years. Of 30 patients with complete patients’ chart, the mean drooling score fell from 7.59 to 2.71 after surgery (p < 0.0001). In 30 patients, results of operation were ascertained by telephone at average of 5.6 years after operation. In 78.1% of patients, long-term result was successful and none were considered worse after the procedure. There were few complications, none of which had any long-term adverse effects. Swelling of submandibular glands was frequently observed in the immediate post-operative period. Only one ranula was seen as delayed complication.

**CONCLUSIONS:**

Submandibular duct relocation with simultaneous sublingual gland excision is a safe and consistently efficient procedure for the treatment of chronic sialorrhea. It is believed that this operation is more physiological procedure than others.

Sialorrhea or drooling is the involuntary passive spillage of saliva out of the mouth. It is a common clinical problem among neurologically impaired children and adults.

Approximately 1.5 L of saliva is secreted into the oral cavity each day. Submandibular glands produce 70% of the resting salivary secretions. Clinically, it is the viscid saliva produced by the submandibular (and sublingual) glands that is the problematic saliva in the drooling child. In contrast parotid secretions are thin and serous.^1^

The causes of drooling are multiple. Persistent drooling in the patients may be the result of either neuromuscular dysfunction (more common) or hypersecretion of saliva. Typically there is a defect in the oral phase of the swallowing, resulting in pooling of saliva in the mouth and eventual spillover. This is brought about by a combination of poor head control, an inability to close the mouth, poor lip control, disordered tongue mobility and a reduction in intraoral tactile sensation.^2^

Drooling is a relatively common clinical sign. According to Tahmassebi’s study, 58% of children with cerebral palsy have a drooling condition, which is severe in 33% of them.^3^ Crysdale estimated that cerebral palsy occurs in 1 of 300 newborns and 10-15% of children with cerebral palsy have significant drooling.^4^–^6^ Also it is seen in many patients with muscular disease.^7^

The unpleasant nature of drooling and salivary spraying while talking can result in the emotional problems and social and psychological isolation. Also drooling causes dermatologic discomfort over the chin and lower face. The inability to swallow adequately increases the risk of aspiration pneumonia.^1^

Many approaches have been used to diminish the amount of drooling, including behavior modification, medical regimens and surgical intervention. Surgical correction of chronic sialorrhea has proved to be the best solution. Several procedures have been advocated. However, some of the procedures maybe associated with significant complications or only a short-term solution.^8^,^9^

Surgical approaches include parotid duct rerouting or ligation, submandibular gland excision, submandibular gland duct rerouting or ligation, sublingual gland excision and division of the parasympathetic nerve supply to the glands.^10^–^17^ Because the submandibular glands produce approximately 70% to 80% of the resting salivary output^4^,^18^ and the aim of surgery is to stop drooling while at the same time maintain a moist healthy oral environment, submandibular duct relocation and sublingual resection (SDRSGE) has been preferred at the Drooling Clinic of Amiralmomenin Hospital since 1994.

The present policy of this clinic is comprehensive assessment of an otolaryngologist, a dentist, a paediatrician, a neurologist, a physiotherapist and a speech pathologist. At first an adequate trial (usually for a minimum of 6 months) of physiotherapy or pharmacotherapy would be started (including anticholinergic or antihistaminic drugs). If trying to provide adequate control of salivary flow failed, surgical procedures would be considered. Also surgical procedure is advised for patients with profuse and constant drooling and those with severe cognitive impairment.

The objectives of this study were to present recommended surgical technique and to assess its complications and to evaluate short and long term result f SDRSGE.

## Methods

Since April 1994 to December 2007 submandibular duct relocation and sublingual resection (SDRSGE) was performed on 32 patients at the Drooling Clinic of Amiralmomenin Hospital, Rasht.

The patients’ charts were retrospectively reviewed to determine following data: preoperative levels of sialorrhea, the overall neurologic status of the patient at the time of operation, operative and postoperative complications, length of postoperative hospitalization, and postoperative levels of sialorrhea after third months.

Preoperative and postoperative levels of sialorrhea were assigned based on the standard scale (Table 1) that was introduced by Crysdale and White in 1989.^17^ Total score is derived by adding the severity score to the frequency score for a total out of 10. Children were reviewed by the multidisciplinary team at 3 months and drooling score was ascertained. Data was available for 30 of the 32 patients.

**Table 1:** Standardized scoring system used to assess drooling severity and frequency

Severity		Frequency	
1. Dry	Never drools	1. Dry	Never drools
2. Mild	Only lips wet	2. Occasional drools	Not every day
3. Moderate	Lips and chin wet	3. Frequent drools	Every day
4. Severe	Clothing	4. Constant drools	All day
5. Profuse	Clothing and tray	5. Constant drools	Wet pillow in AM

The overall neurologic status of the patient was ascertained by oral function (the patient’s ability to articulate and to swallow). Developmental neuromuscular control was estimated by assessing the amount of head control and the ability to walk. Overall neurologic status was gauged as mild (within normal limits), moderate (some deviation such as weakness or inconsistency of movement), or severe (unable to perform the task).

The parents of the patients were contacted by telephone at least one year after operation. The parents were questioned about success of surgical treatment and health status of patients (dental caries, xerostomia, lower respiratory tract infection, etc). The success of surgical treatment was assessed with the criteria described by Wilkie and Brody^6^: long term result is considered “excellent” if there is no drooling but lower lip may be moist, “good” if there is saliva on chin and drooling less than once each day, “fair” if there is saliva on chin and drooling at least once each day, and “poor” if no significant control is observed. Successful surgical removal includes procedures with “excellent” or “good” results.

Statistical analysis was performed using SPSS 13.0 and the significance level was set at 0.05 for all tests. Wilcoxon signedrank tests were used to compare preoperative and postoperative drooling scores.

### 

#### Operative Technique

In all patients the operation was performed under a general anesthesia through an orotracheal tube. At first a tonsillectomy was performed, if they are large. Then the tongue was retracted out of the field of dissection using a 0 silk suture placed in the tip of the tongue and soft palate. The opening of the submandibular duct in the floor of the mouth with a surrounding cuff of mucosa was incised and duct was identified and skeletonized of their surrounding tissue by sharp dissection without the use of cannulation. The duct was dissected back to the lingual nerve and approximately 3 to 4 cm of duct was released. Then a submucosal tunnel was created through the floor of the mouth.

The exit site of tunnel was created near the anterior pillar of tonsillar fossa. The submandibular duct was then passed through the tunnel and secured to the anterior tonsillar pillar with only a single 4.0 vicryl stitch. In this operation, ductules of the sublingual glands were transected as they entered the submendibular ducts. Thus sublingual resection was performed and the donor site of mucosal island was repaired. It is crucial to avoid the large veins in the floor of the mouth as ligation of those veins may result in tremendous tongue swelling. This operation was essentially by Crysdale.^19^ Originally Crysdale performed a routine tonsillectomy before duct relocation, in order to prevent retrograde sialadenitis secondary to tonsillitis.

## Results

The medical records of all children who underwent SDRSGE (n = 32) were available for review. Of 32 patients were treated with SDRSGE, 14 were girls (43.7%) and 18 were boys (56.3%). The ages at surgery ranged from 6 to 26 years old. Overall neurologic status of 13 patients was mild. Majority of the patients (50%) had mental handicap. Eleven were affected by cerebral palsy, four were mentally retarded owing to an unknown cause, and one had neurosurgery operation. Epilepsy was particularly common (37.5%).

The average length of hospital stay was 4 days (range: 2-5 days). There were no operative complications. Swelling of submandibular glands was frequently observed in the immediate postoperative period. It was rarely symptomatic and usually subsided within the first week. In all cases the swelling settled spontaneously and did not require active intervention. There was no lateral cervical cyst formation that is believed to arise from obstruction of the relocated duct. Ranula occurred in one patient 3 months following the procedure, requiring surgical resection. There were no other complications.

The mean presurgical score was 7.59 (range: 5 to 10) and the mean postoperative score was 2.71 (range: 2 to 5) which represents a change from severe constant drooling to mild, moderate and occasional drooling. This change was evaluated with the Wilcoxon signed rank test and found to be significant to a p value less than 0.0001. The families of 30 patients (93.75%) were interviewed. The average time of followup was 5.6 years (range: 1-13 years). Long term result of the procedure in 25 patients (78.1%) was successful (Table 2). All the families reported the long- term postoperative improvement in drooling. None of those reported dental caries, xerostomia or lower respiratory tract infections after the surgery.

**Table 2:** Long term result of SDRSGE

Outcome	Number (%)
Excellent	17 (53.1)
Good	8 (25%)
Fair	5 (15.6%)
Poor	-
Unknown	2 (4.9%)
Total	32 (100%)

## Discussion

It is well known that transposition of submandibular duct to the posterior part of the oral cavity help to swallow secretions by gravity. Bilateral submandibular duct transposition alone was first reported by Laage-Hellmann in 1969 and adopted by others.^2^,^8^,^17^ Crysdale performed bilateral submandibular duct transposition without bilateral sublingual gland excision,^17^ reported good or excellent results in 67 percent of 194 patients with at least 1-year follow up but 8 percent developed ranulae arising from the sublingual glands. Ranulas develop as extravasation pseudocysts arising from disrupted sublingual gland tissue. Although adding simultaneous sublingual gland excision adds significantly to the duration of the surgery, rate occurrence of ranula and lateral neck cyst decreased.^9^ More recently, Crysdale reported improved results and less complication with the addition of routine sublingual gland excision to bilateral submandibular duct transposition.^9^

Although an increase in the prevalence of caries after submandibular duct relocation has been reported,^20^ no increase in caries was found in the present study. Xerostomia has not been documented following presented procedure. Although the increased salivary flow to the oropharynx may cause salivary contamination of the respiratory tree,^21^ no patient had an increased number of respiratory infections. The long term success rate of SDRSGE has been good in our study (78.1%) that is similar to all reported studies, ranging from 79.6% to 92%.^2^,^21^–^23^ Surgery rarely eliminates drooling so most patients can except to have residual drooling because of situational factors that cannot be eliminated and continuing oral-motor dysfunction. The only children who may have a perfect result are those likely to have xerostomia.

Because the maturation of oral-motor function in children with cerebral palsy is delayed until age 5 or 6, the operation in these patients was deferred until at least this age.^24^

## Conclusions

Although there are several procedures for surgical control of drooling, this report demonstrates that SDRSGE is a safe and highly effective procedure to control excessive drooling. It is associated with minimal morbidity and favorable result in long term. Other advantages include technical ease, shorter anesthetic time and lack of external scars. Present study also demonstrated that this procedure improves the quality of life of patients.

## References

[1] Lal D, Hotaling AJ (2006). Drooling. Curr Opin Otolaryngol Head Neck Surg.

[2] O’Dwyer TP, Conlon BJ (1997). The surgical management of drooling: a 15 year follow- up. Clin Otolaryngol Allied Sci.

[3] Tahmassebi JF, Curzon ME (2003). Prevalence of drooling in children with cerebral palsy attending special schools. Dev Med Child Neurol.

[4] Hussein I, Kershaw AE, Tahmassebi JF, Fayle SA (1998). The management of drooling in children and patients with mental and physical disabilities: a literature review. Int J Paediatr Dent.

[5] Martin TJ, Conley SF (2007). Long term efficacy of intra-oral surgery for sialorrhea. Otolaryngol Head Neck Surg.

[6] Crysdale WS, McCann C, Roske L, Joseph M, Semenuk D, Chait P (2006). Saliva control issues in the neurologically challenged: a 30 year experience in team management. Int J Pediatr Otorhinolarygol.

[7] Harris SR, Purdy AH (1987). Drooling and its management in cerebral palsy. Dev Med Child Neurol.

[8] Ekedahl C (1974). Surgical treatment of drooling. Acta Otolaryngol.

[9] Crysdale WS, Raveh E, McCann C, Roske L, Kotler A (2001). Management of drooling in individuals with neurodisability: a surgical experience. Dev Med Child Neurol.

[10] Wilkie TF, Brody GS (1977). The surgical treatment of drooling. A ten-year review. Plast Reconstruct Surg.

[11] Arnold HG, Gross CW (1977). Transtympanic neurectomy: a solution to drooling problems. Dev Med Child Neurol.

[12] Frederick FJ, Stewart IF (1982). Effectiveness of transtympanic neurectomy in management of sialorrhea occurring in mentally retarded patients. J Otolaryngol.

[13] Mullins WM, Gross CW, Moore JM (1979). Long- term followup of tympanic neurectomy for sialorrhea. Laryngoscope.

[14] Guerin RL (1979). Surgical management of drooling. Arch Otolaryngol.

[15] Dundas DF, Pererson RA (1979). Surgical treatment of drooling by bilateral parotid duct ligation and submandibular gland resection. Plast Reconstr Surg.

[16] Glass LW, Nobel GL, Vecchione TR (1978). Treatment of uncontrolled drooling by bilateral excision of submaxillary glands and parotid duct ligations. Plast Reconstr Surg.

[17] Crysdale WS, White A (1989). Submandibular duct relocation for drooling: a 10-year experience with 194 patients. Otolaryngol Head Neck Surg.

[18] Stuchell RN, Mandel ID (1988). Salivary gland dysfunction and swallowing disorders. Otolaryngol Clin North Am.

[19] Crysdale WS (1980). The drooling patient: evaluation and current surgical options. Laryngoscope.

[20] Arnrup K, Crossner CG (1990). Caries prevalence after submandibular duct retroposition in drooling children with neurological disorders. Pediatr Dent.

[21] Mankarious LA, Bottrill ID, Huchzermeyer PM, Bailey CM (1999). Long- term followup of submandibular duct rerouting for the treatment of sialorrhea in the pediatric population. Otolaryngol Head Neck Surg.

[22] Meningaud JP, Pitak- Arnnop P, Chikhani L, Chikhani L, Bertrand JC (2006). Drooling of saliva: a review of the etiology and management options. Oral Surg Oral Med Oral Pathol Oral Radiol Endod.

[23] Greensmith AL, Johnstone BR, Reid SM, Hazard CJ, Johnson HM, Reddihough DS (2005). Prospective analysis of the outcome of surgical management of drooling in the pediatric population: a 10-year experience. Plast Reconstr Surg.

[24] Bluestone CD, Stool SE, Alper CM, Arjmand EM, Casselbrant ML, Dohar JE (2001). The management of drooling. Pediatric Otolaryngology.

